# Perceptions of immunisation adherence by caregivers in the Western Cape province, South Africa: A qualitative study

**DOI:** 10.4102/curationis.v49i1.2888

**Published:** 2026-07-10

**Authors:** Bridgette Lockett, Juliana J. Willemse

**Affiliations:** 1 School of Nursing, Faculty of Community & Health Sciences, University of the Western Cape, Cape Town, South Africa

**Keywords:** adherence, barriers, caregivers, immunisation, perceptions

## Abstract

**Background:**

Although the Expanded Programme on Immunisation in South Africa has received considerable funding for universal access, immunisation rates remain low. It is important to understand the perceptions of caregivers to develop interventions that can improve immunisation adherence.

**Objectives:**

To examine the perceptions of caregivers in relation to immunisation adherence in the selected Western Cape districts, and to examine the important barriers and facilitators that affect the immunisation process in urban primary healthcare.

**Method:**

A qualitative research method was employed to gain a clear understanding of the phenomenon. The semi-structured interview technique was employed using an interview guide focused on the perceptions of caregivers regarding immunisation adherence. The participants were selected through purposive and convenience sampling. The qualitative thematic analysis technique was employed to identify themes based on the framework proposed by Braune and Clark, as discussed in the data analysis and management section of this article.

**Results:**

Six themes emerged that affect immunisation compliance: barriers to accessibility, communication, efficiency of the healthcare system, knowledge and attitudes of caregivers, socioeconomic factors, and the role of cultural and religious beliefs.

**Conclusion:**

The conclusion of the study was that caregivers showed high levels of commitment to childhood immunisation, but were hindered in their efforts by systemic, socioeconomic and cultural factors.

**Contribution:**

The contribution of the study is that it offers evidence that can be used to develop strategies to improve immunisation compliance.

## Introduction

Immunisations represent one of the most cost-effective public health interventions, preventing millions of deaths annually and contributing significantly to child survival and development (United Nations Children’s Fund [UNICEF] 2023; World Health Organisation [WHO] 2023). However, despite global progress in immunisation coverage, substantial disparities persist, particularly in low- and middle-income countries (LMICs), where access barriers, health system constraints and inequities continue to impede optimal immunisation outcomes (Gavi 2022; WHO 2023). South Africa, despite substantial investments to ensure universal access to immunisation services through the Expanded Programme on Immunisation (EPI), continues to face challenges in achieving and sustaining optimal coverage across provinces and districts, particularly in underserved communities (National Department of Health [NDoH] 2023; UNICEF South Africa 2023). Recent evidence from African contexts further demonstrates that immunisation adherence is influenced by complex and interrelated caregiver, socio-cultural and health system factors, including vaccine confidence, service accessibility and provider communication (Wiysonge et al. 2022; WHO 2023).

Research conducted LMICs and within the African contexts has identified multiple barriers to immunisation adherence, including limited vaccine knowledge, concerns about vaccine safety, logistical challenges such as transport and clinic waiting times and gendered decision-making dynamics that constrain caregivers’ autonomy in completing immunisation schedules (Wiysonge et al. 2022; WHO 2023). Understanding these multilevel determinants is essential for designing responsive, context-specific interventions that strengthen vaccine uptake and completion rates (Gavi 2022; UNICEF 2023).

The Western Cape province of South Africa presents a distinctive context for examining immunisation adherence. While characterised by relatively well-developed urban healthcare infrastructure and established primary healthcare services, significant socioeconomic disparities persist, which continue to influence healthcare access, continuity of care and service utilisation patterns (NDoH 2023; Western Cape Department of Health and Wellness 2023). Although existing literature underscores the importance of caregiver perceptions, trust in health services and lived experiences in shaping immunisation decisions, there remains limited qualitative research that deeply explores these contextualised perceptions within the contemporary South African setting (UNICEF South Africa 2023; Wiysonge et al. 2022).

This study was guided by the Behavioural and Social Drivers (BeSD) of Vaccination framework developed by the WHO, which conceptualises immunisation behaviour as the result of interacting influences at individual, interpersonal, community and health system levels (WHO 2023). The BeSD model emphasises four interrelated domains shaping vaccination decisions: caregivers’ thoughts and feelings about vaccines, confidence and risk perception; social processes that influence decision-making, including norms and trust; motivation or intention to vaccinate; and practical issues that enable or constrain access, such as service availability and convenience (UNICEF 2023; WHO 2023). Recognising the complexity of immunisation decision-making and the documented influence of contextual and systemic determinants in African settings (Wiysonge et al. 2022), this study sought to generate context-specific insight within the Western Cape province of South Africa, where socioeconomic inequalities and diverse cultural contexts may influence vaccine compliance (National Department of Health [NDoH] 2023).

Accordingly, the study aimed to explore caregivers’ perceptions of immunisation adherence, identify key barriers and enablers, examine caregivers’ interactions with healthcare services during immunisation visits, and elucidate the role of socioeconomic, cultural and communication factors in vaccination decision-making. The findings are intended to inform the development of evidence-based, equity-focused interventions to strengthen immunisation compliance in urban South African primary healthcare settings.

### Rationale of the study

Immunisation coverage has been observed to stagnate at a concerning rate in recent years, with rising numbers of zero-dose and under-immunised children worldwide, especially in LMICs (UNICEF 2023; WHO 2023). In South Africa, while the EPI offers universal access through primary healthcare services, there are still variations in coverage at a provincial and district level (NDoH 2023). Routine administrative statistics measure these gaps but fail to capture the contextual and experiential aspects of factors that influence caregiver decision-making and service use. Recent evidence from the African continent indicates that vaccine acceptance is a complex phenomenon that is mediated by a range of intersecting social, behavioural, and health system factors, such as trust, communication, and structural barriers to access (Wiysonge et al. 2022). Nonetheless, there is currently a lack of context-specific research examining the role of these factors within the Western Cape’s primary healthcare context.

### Problem statement

Despite the availability of immunisation services through South Africa’s EPI, variations in vaccine compliance persist across provinces and communities (NDoH 2023). While routine administrative data quantify coverage levels, they provide limited insight into the contextual and behavioural factors influencing caregivers’ immunisation decisions. Emerging evidence highlights that vaccine uptake is shaped by complex interactions between caregiver perceptions, social influences and health system factors (Wiysonge et al. 2022; WHO 2023). However, there is limited context-specific qualitative research exploring how these determinants influence immunisation compliance within the Western Cape Primary Healthcare setting. This evidence gap constrains the development of targeted, equity-sensitive strategies aimed at strengthening vaccine uptake. This study therefore explored caregivers’ perceptions of immunisation compliance in the Western Cape to inform responsive, evidence-based interventions.

### Research methods and design

A qualitative descriptive research design was employed in this study to obtain in-depth data about the participants’ perceptions in relation to immunisation adherence. The application of a qualitative descriptive research design is particularly important when the aim of the research is to develop a detailed description of the participants’ perspectives, meanings and interpretations of a phenomenon in everyday language (Polit & Beck 2024). This research design allowed for a detailed exploration of the caregivers’ perceptions of immunisation services, including their understanding, interpretation and evaluation of factors that affect immunisation adherence in their everyday healthcare environment (Brink & Van Rensburg 2022). By exploring perceptions rather than measuring predefined variables, the study was able to conduct a detailed exploration of the participants’ perspectives on how the caregivers interpret immunisation practices and decision-making processes (Creswell & Creswell 2023).

### Setting

The research was carried out at selected public primary healthcare facilities in the City of Cape Town Health Districts in the Western Cape province, South Africa. The study was carried out in six health districts that include a wide range of socioeconomic settings, from highly populated urban areas to peri-urban areas. These health districts are part of the primary healthcare system administered by the province, which is charged with the responsibility of providing routine child health and immunisation services to diverse groups of people.

The Western Cape province is known to have a high degree of geographical and socioeconomic variation, which influences the utilisation of healthcare services. Most caregivers rely on public transportation like minibus taxis, buses and trains to access healthcare services. Transport-related factors, particularly in the winter rainfall region of the province during adverse winter weather conditions, may influence the time, cost and convenience of accessing healthcare services at clinics. Geographical and transport-related factors may function through several mechanisms, including distance to health facilities, absence of public transportation, weather conditions, time constraints due to employment and caring roles and cost of accessing care.

### Population

The target population was caregivers of children who were accessing routine immunisation services at selected public primary healthcare facilities in the Western Cape province, South Africa. Caregivers were selected as participants for the study because they were important decision-makers who ensured that children strictly adhered to immunisation schedules and were thus directly positioned to provide information on factors that influence immunisation schedules. Healthcare providers at the selected facilities acted as gatekeepers who introduced the study to caregivers who met the predetermined criteria. This ensured that ethical access to participants was provided while maintaining confidentiality and avoiding the possibility of coercion. Caregivers who expressed interest were referred to the researcher for more information about the study.

### Sampling

The sampling area consisted of purposively sampled public primary healthcare facilities in the Western Cape province in South Africa. Participants for the study were caregivers of children attending routine immunisation services, as they are the key decision-makers responsible for ensuring that children adhere to scheduled immunisations.

### Sample size

The sample size was calculated using the principle of data saturation. Semi-structured interviews were carried out on 16 caregivers who had recent experience with childhood immunisation services. Data gathering was done simultaneously with data analysis, which enabled the continuous identification of themes emerging from the interviews. Recruitment of participants continued until no new themes or meaningful information could be derived from a series of interviews, which indicated that thematic saturation was attained. The lack of new information in the final interviews indicated that a sufficient level of depth, diversity and richness of information had been gathered to satisfactorily address the research aims. This ensured methodological rigor and facilitated the credibility and completeness of the results (Brink & Van Rensburg 2022).

### Pretest

The pretest was carried out using two caregivers who were recruited from two of the purposefully selected public primary healthcare facilities in the Western Cape province. These participants fulfilled the inclusion criteria but were not included in the final study sample to avoid contaminating the primary data set. Excluding pilot study participants from the primary analysis is recommended to maintain methodological rigor in qualitative studies (Polit & Beck 2024). The aim of the pretest was to determine the appropriateness, clarity and sensitivity of the semi-structured interview questions, especially in terms of the participants’ willingness to respond to questions about their perceptions of immunisation services. Pilot testing is considered crucial in qualitative research to determine the understanding of the questions, cultural acceptability and the effectiveness of the probing techniques (Creswell & Creswell 2023). The pilot test allowed the researcher to assess if there were any questions that caused discomfort, ambivalence, or ambiguity to the participants and to determine the extent of communication clarity and rapport-building needed in the healthcare setting. The interview schedule was not altered after the pretest as the pilot test was successful in meeting its research objectives and confirmed the appropriateness of the research instrument.

### Data collection

Data were collected using in-depth, semi-structured interviews guided by the BeSD model and literature related to immunisation acceptance. This allowed for the exploration of participants’ perceptions, barriers, socioeconomic and cultural factors, and suggestions for service improvement (Creswell & Creswell 2023; Polit & Beck 2024). Semi-structured interviews were conducted face to face between 01 June 2024 and 31 December 2024 in the participants’ language of choice (English, Afrikaans, or isiXhosa) by trained multilingual research assistants to improve understanding and validity (Brink & Van Rensburg 2022). All interviews were recorded with participants’ consent prior to the start of data collection, transcribed verbatim, and, if necessary, translated into English using back-translation methods to ensure semantic meaning accuracy and equivalence (Creswell & Creswell 2023). Confidentiality was maintained by anonymising transcripts and analysis documents, assigning each participant a unique identifier, and removing any information that could identify participants, thus protecting participants’ privacy and adhering to ethical standards.

### Data analysis and management

Qualitative data were analysed using Braun and Clarke’s (2006, 2022) six-step reflexive thematic analysis approach. All interviews were audio-recorded with consent and transcribed verbatim to ensure accuracy and preserve participants’ original meanings. Transcripts were checked against recordings for completeness and anonymised prior to analysis. Data were stored securely in password-protected electronic files accessible only to the research team, in accordance with ethical requirements. An inductive analytical approach was employed to allow themes to emerge directly from participants’ accounts rather than being imposed a priori (Braun & Clarke 2022).

After translation into English, analysis began with familiarisation, during which transcripts were read and re-read to gain an in-depth understanding of the content, while initial analytic notes were recorded. In the second phase, systematic coding was conducted across the dataset to identify meaningful units of text reflecting patterns, concepts and recurrent ideas. Codes were then organised into potential themes through a process of collating and reviewing patterns of association. During the fourth phase, themes were reviewed against the coded extracts and the full dataset to ensure coherence, consistency and conceptual distinctiveness.

Themes were refined iteratively, with peer debriefing sessions conducted to enhance analytic rigour and reflexivity. In the fifth phase, themes were clearly defined and named, ensuring that each theme captured a central organising concept grounded in the data. The final phase involved producing the report, integrating analytic insights with illustrative participant quotations to support transparency and credibility. Data collection and analysis occurred concurrently, and saturation was considered achieved when subsequent interviews yielded no new codes or thematic insights, indicating sufficient depth and completeness of the dataset (Creswell & Creswell 2023; Polit & Beck 2024). This systematic and reflexive process enhanced the credibility, dependability and confirmability of the findings.

### Measures to ensure trustworthiness

Informed by the framework of trustworthiness proposed by Lincoln and Guba (1985), this study ensured rigour in terms of credibility, transferability, dependability and confirmability.

*Credibility* was enhanced by prolonged engagement with participants after the conduct of semi-structured interviews, use of multiple sources of data (carers included), verbatim transcription of digitally recorded interviews and keeping an audit trail.

*Transferability* was facilitated by the use of rich and detailed descriptions of participants and the research context, member checking of transcripts and independent coding of themes and sub-themes to arrive at a consensus.

*Dependability* was ensured by systematic documentation of the research process and validation of findings by an independent coder, thus ensuring consistency in analysis.

*Confirmability* was ensured by independent validation of data interpretation and the use of reflective practices, where the researcher recognised the role of their own perspectives in the research process.

Inductive analysis facilitated the development of themes from the data, and deductive reasoning and triangulation helped to ensure that the results of childhood immunisation adherence were credible, transparent and morally sound.

### Ethical considerations

Ethical principles were integrated throughout the study, in line with the Declaration of Helsinki, and ethical clearance to conduct this study was obtained from the Biomedical Science Research Ethics Committee of the University of the Western Cape (No. BM23/10/1) and the Western Cape Department of Health.

The research was carried out based on the well-known ethical standards that have been set out in the Belmont Report and guidelines provided by the South African Nursing Council, which include respect for persons, beneficence, and justice, thereby protecting the rights, dignity and welfare of the participants.

*Respect* for persons was shown by providing participants with all necessary information and securing their informed written consent so that participants would agree voluntarily to take part in the study while having an option to leave whenever they wanted to, without facing any repercussions.

*Beneficence* was observed by reducing risks of any kind, conducting the interview process in an encouraging way, and informing participants about the lack of any benefit from being involved in the study other than possible improvement in healthcare practices.

*Justice* was ensured through fair participant selection based on clear inclusion criteria and equitable treatment throughout the study.

*Anonymity and confidentiality* were ensured by using coded transcripts, storing password-protected and encrypted data for 5 years, and allowing access only to authorised persons. The study did not pose any risk to participants and was intended to contribute to the development of a support system to improve adherence to the immunisation schedule.

## Results

The research offers the results of 16 semi-structured interviews carried out in the chosen districts of the Western Cape province. In-depth semi-structured interviews carried out in private settings in healthcare facilities yielded rich qualitative information. Semi-structured interviews were carried out in English, with Afrikaans and isiXhosa translation if required, audio-recorded with permission, transcribed verbatim, anonymised and safely archived. Field notes added depth to the transcripts by providing contextual and non-verbal information for enhanced interpretation.

The thematic analysis yielded six key themes and twelve consolidated subthemes (Table 1) that clarify caregivers’ perceptions of immunisation adherence in the chosen districts of the Western Cape province.

**TABLE 1:** Themes and subthemes.

Main theme	Subthemes
1. Accessibility barriers in immunisation services	1.1: Transportation and geographic challenges 1.2: Work schedule, safety and environmental barriers
2. Communication and information	2.1: Health literacy and immunisation information needs 2.2: Digital communication preferences
3. Healthcare system efficiency	3.1: Waiting times and service delivery processes 3.2: Appointment systems and supply chain management
4. Parent attitudes and knowledge	4.1: Perceived importance of immunisation and provider trust 4.2: Vaccine hesitancy and misinformation impact
5. Socioeconomic factors	5.1: Financial constraints and cost-related access barriers 5.2: Safety in neighbourhood areas 5.3: Work-related barriers
6. Cultural and belief systems	6.1: Traditional beliefs and cultural practices 6.2: Medical mistrust and community influence

The results are presented here, using the refined subtheme framework, with supporting quotations to give voice to the participants.

### Main theme 1: Accessibility barriers in immunisation services

Caregivers described immunisation access as shaped by intersecting physical, logistical and contextual constraints. Barriers included transport and distance, poor infrastructure, adverse weather, rigid clinic hours that conflicted with work demands and community safety concerns. Collectively, these factors disrupted timely clinic attendance and contributed to missed or delayed immunisation visits, consistent with evidence that access constraints strongly influence immunisation coverage in comparable settings (Martines & Kumar 2024; Thompson, Chen & Wilson 2023).

The accessibility of immunisation was described as influenced by intersecting physical, logistical and contextual factors. Barriers included transport and distance, poor infrastructure, inclement weather, inflexible clinic times that did not align with work commitments and safety concerns in the community. These factors, taken together, impacted clinic attendance and contributed to non-attendance or delayed immunisation visits, as has been found to influence immunisation coverage in similar contexts (Martines & Kumar 2024; Thompson et al. 2023).

#### Subtheme 1.1: Transportation and geographic challenges

Transport problems were also cited as a major challenge, especially for caregivers who could not afford transport or relied on public transport routes. The participants highlighted how frequent visits to the clinic made the problem worse:

‘I didn’t have that transport to always go to the clinic.’ (Participant 8, 39 years old, female, unemployed)

The long distances involved in walking with babies, especially during cold or rainy weather, were also considered physically demanding and hazardous. These perceptions are consistent with the South African study that found transport and distance to be major predictors of immunisation delays (Wiysonge et al. 2021), as well as the evidence that increasing distance to facilities is associated with decreased uptake (Ndwandwe et al. 2021; Wilson & Park 2024).

#### Subtheme 1.2: Work schedule, safety and environmental barriers

Apart from distance, the operating hours of the clinics were also cited as sometimes conflicting with the work commitments of caregivers, making it difficult for them to visit the clinics during working hours:

‘Then I won’t be able to go on that day. Sometimes you get one day.’ (Participant 4, 31 years old, male, construction worker)

‘It’s a bit tricky when you have to work and visit the clinic.’ (Participant 10, 29 years old, female, domestic worker)

The early morning operating hours of the clinics were also cited as contributing to the effects of winter weather on those who walked to the clinics:

‘They must schedule it a little bit later… it’s rainy, it’s cold … especially with newborn babies.’ (Participant 2, 27 years old, female, unemployed)

‘7 o’clock is way too early … maybe from 8 o’clock … or 9 o’clock.’ (Participant 1, 24 years old, female, unemployed)

Safety concerns, particularly gang violence, were cited as making it difficult for caregivers to visit the clinics with babies:

‘Because when there are gangsters, people don’t take their kids for immunisation … difficult to travel and to move.’ (Participant 11, 34 years old, female, unemployed)

Such results are in line with the literature that has shown the existence of ‘invisible boundaries’ in the provision of care when insecurity and risk are taken into account (Cooper, Wiysonge & Ndwandwe 2020), as well as the effectiveness of care models that take into account local conditions (such as extended hours of operation) in improving access and adherence (Burnett et al. 2019; Ndwandwe et al. 2021).

### Main theme 2: Communication and information

Facilities’ communication experiences affected caregivers’ engagement, trust and intention to return for a follow-up dose. Participants reported both interpersonal communication challenges, including tone, respect and clarity, and system-level communication gaps. As supported by previous evidence, poor communication affected trust and adherence, while respectful and clear communication supported the completion of immunisation schedules (Thompson et al. 2023; Wiysonge et al. 2021).

#### Subtheme 2.1: Health literacy and immunisation information needs

Caregivers often reported a lack of explanation regarding vaccines, benefits and side effects, as if the process was more procedural than educational:

‘No, they don’t explain. They just give the shot.’ (Participant 14, 37 years old, female, domestic worker)

Some caregivers reported a lack of time or resources to decode written information:

‘I don’t have time to read the book. So, they don’t make time to explain.’ (Participant 14, 37 years old, female, domestic worker)

Others reported that when an explanation was available, it enhanced understanding and confidence:

‘So, I’m just doing what I’m supposed to do with my second one … she explains that’s what’s important.’ (Participant 4, 31 years old, male, construction worker)

Participants also expressed a desire for open, useful information about each vaccine:

‘What sickness … what is it going to keep away?.’ (Participant 5, 28 years old, female, unemployed)

These results indicate that a lack of health literacy support and poor counselling can limit well-informed compliance, supporting the importance of culturally and linguistically valid education (Ndwandwe et al. 2021; WHO 2023).

#### Subtheme 2.2: Digital communication preferences

A clear preference was seen for reminder systems and electronic record choices to improve attendance and access to information. Suggestions were made for reminder systems via SMS:

‘Maybe if you can get a reminder day or two before the time.’ (Participant 10, 29 years old, female, domestic worker)

‘If you have at least an SMS … it will be better.’ (Participant 2, 27 years old, female, unemployed)

Others suggested the digitisation of the Road-to-Health card:

‘Instead of a book … you can have a digitalised book.’ (Participant 11, 34 years old, female, unemployed)

These opinions support the use of digital reminder systems to improve timeliness and adherence (Martines, Kumar & Chen 2023; Park & Rodrigues 2024), and the use of digital strategies to support person-centred communication and prevent the exacerbation of inequities (WHO 2021).

### Main theme 3: Healthcare system efficiency

Inefficiencies in the system were mentioned as barriers that worked against frequent visits to the clinic. Service delays, personnel availability, lack of consistency in workflow, scheduling and stockouts were associated with frustration, lost work time and decreased confidence in the reliability of services, which is consistent with the literature that associates service delivery with immunisation coverage (Thompson et al. 2023; Wiysonge et al. 2021).

#### Subtheme 3.1: Waiting times and service delivery processes

Waiting for a long time was one of the common issues raised. Some participants indicated that they waited for a few hours to access one immunisation service:

‘You come 9 o’clock, but you might walk out 4 o’clock.’ (Participant 10, 29 years old, female, domestic worker)

‘Sometimes you spend four or five hours … just to receive one injection.’ (Participant 11, 34 years old, female, unemployed)

Waiting was also associated with the availability of staff:

‘Maybe have more staff who can help with immunisation only.’ (Participant 10, 29 years old, female, domestic worker)

The above statements demonstrate how inefficiencies result in opportunity costs (time, money and childcare), which may act as a deterrent for follow-up attendance (Ndwandwe et al. 2021).

#### Subtheme 3.2: Appointment systems and supply chain management

Caregivers highlighted the possible benefit of organised appointment systems to alleviate time constraints and facilitate working caregivers:

‘If your appointment is 10 o’clock and you get by 10 o’clock, you can still return to work.’ (Participant 12, 33 years old, female, domestic worker)

However, vaccine stockouts and communication breakdowns regarding follow-up were also cited as demotivating and stressful:

‘When I arrived … I didn’t get that injection.’ (Participant 4, 31 years old, male, construction worker)

‘They said they would call me back … yet never called me.’ (Participant 5, 28 years old, female, unemployed)

One caregiver mentioned that stockouts caused feelings of guilt and uncertainty:

‘… it made me think like now, I’m being negligent.’ (Participant 7, 28 years old, female, self employed)

These results underscore the importance of both organised appointment systems and organised supply systems to ensure continuity and trust (Burnett et al. 2019; WHO 2021).

### Main theme 4: Parent attitudes and knowledge

Caregivers’ beliefs about immunisation, together with trust and exposure to misinformation, influenced adherence choices. Although many caregivers strongly adhered to immunising their children, others reported community-level levels of doubt and fear that contributed to hesitancy. These findings reflect the importance of attitudes, trust and information environments in vaccine uptake, as supported by evidence (Larson et al. 2020; Wilson & Kumar 2024).

#### Subtheme 4.1: Perceived importance of immunisation and provider trust

Many caregivers considered immunisation a non-negotiable parental duty: ‘If your child’s injection date… you must attend the clinic… it’s for your child’s health’. (Participant 5, 28 years old, female, unemployed) and ‘I have a date, so I have to go for the injection’. (Participant 7, 28 years old, female, self employed). However, the attitudes and interpersonal behaviours of the providers influenced trust and comfort levels in interacting with the services:

‘Most times, nurses are a little too harsh.’ (Participant 1, 24 years old, female, unemployed)

‘It was about the attitude.’ (Participant 11, 34 years old, female, unemployed)

These views indicate that even if caregivers appreciate immunisation, poor experiences can reduce engagement and attendance (Cooper et al. 2020; Wiysonge et al. 2021).

#### Subtheme 4.2: Vaccine hesitancy and misinformation impact

Participants spoke of being exposed to negative community messages that doubted the safety or need for vaccines:

‘People are telling us now the immunisation is not good.’ (Participant 8, 39 years old, female, unemployed)

‘Sometimes people tell negativity about immunisation.’ (Participant 6, 38 years old, female, unemployed)

It is worth noting that some of the caregivers’ spoke of resisting misinformation:

‘… she was giving me negative answers … But I told myself … it’s actually a good thing.’ (Participant 6, 38 years old, female, unemployed)

This shows the vulnerability to misinformation and the presence of individual agency, which emphasises the need for effective communication (Ndwandwe et al. 2021; WHO 2023).

### Main theme 5: Socioeconomic factors

Socioeconomic factors influenced immunisation behaviour by reducing the affordability of transportation, the opportunity cost of time away from work and the effects of unsafe neighbourhood environments. The participants’ voices demonstrate the challenges of ‘free’ services in the context of poverty constraints, which align with evidence on the role of indirect costs in immunisation inequities (Cooper et al. 2020; Martines et al. 2023).

#### Subtheme 5.1: Financial constraints and cost-related access barriers

The caregivers explained how the limited income impacted both the availability of transport and the choice to look for alternative transport:

‘And I cannot go private also because I don’t have the money.’ (Participant 11, 34 years old, female, unemployed)

The cost of transport was cited as a dissuading factor:

‘I didn’t have the transport to always go to the clinic.’ (Participant 7, 28 years old, female, self employed)

These examples show how affordability is a factor through direct and indirect costs including travel, time and childcare, which emphasises the importance of equity-oriented access support in low-income settings (WHO 2021).

#### Subtheme 5.2: Safety in neighbourhood areas

The relationship between socioeconomic vulnerability and unsafe living environments was found to be very close since caregivers highlighted the issue of violence and criminality in their communities that prevented them from accessing any healthcare service in the area. The issue of fear of moving around the neighbourhood appeared to be an important factor affecting access to care, especially when travelling to clinics together with the kids. Thus, for instance, one of the participants claimed:

‘I stay in a very dangerous community.’ (Participant 10, 29 years old, female, domestic worker)

This finding is well in line with the current literature on the subject (Ndwandwe et al. 2021; WHO 2021).

#### Subtheme 5.3: Work-related barriers

Economic constraints due to job pressures posed yet another barrier to accessing healthcare. The informants pointed out that rigid working hours and job losses acted as barriers to accessing clinics. As one participant noted:

‘Production should continue … and therefore it is very hard … because you need to leave the place’. (Participant 2, 27 years old, female, unemployed)

While another informant said:

‘You do not know whether you are going to be at work or not anymore’ (Participant 12, 33 years old, female, domestic worker).

The above quotes illustrate the nature of employment in resource-poor settings, where seeking healthcare would mean putting one’s job security at risk. Thus, barriers to accessing health facilities may be created by workplace issues (Ndwandwe et al. 2021; WHO 2021).

### Main theme 6: Cultural and belief systems

Caregiver interpretations of immunisation were also influenced by cultural norms and intergenerational transmission of beliefs, sometimes supporting and other times undermining adherence. The role of family and community stories in shaping decision-making was mentioned by participants, providing evidence for the importance of culturally informed strategies to achieve sustained uptake (Burnett et al. 2019; Ndwandwe et al. 2021).

#### Subtheme 6.1: Traditional beliefs and cultural practices

The participants mentioned social and family influences that discouraged immunisation and presented it as a risky activity:

‘They say don’t bring your child to immunisation; it will be dangerous.’ (Participant 12, 33 years old, female, domestic worker)

‘It’s the way my mother put in my mind.’ (Participant 13, 37 years old, female, unemployed)

These views indicate the role of belief systems in influencing risk and appropriate care practices, and the need for strategies that respect these systems while conveying biomedical information (WHO 2021).

#### Subtheme 6.2: Medical mistrust and community influence

Lack of trust in healthcare and community-level scepticism were also identified as factors, especially when others in the community demonstrated non-adherence:

‘I’m around young ladies who don’t bring their kids for immunisation.’ (Participant 11, 34 years old, female, unemployed)

Participants also reflected on the impact of community-level claims about the harmfulness of vaccines:

‘People are telling us now the immunisation is not good.’ (Participant 12, 33 years old, female, domestic worker)

However, some caregivers also reported challenging community influence:

‘Heard these negative things … but it didn’t influence me.’ (Participant 7, 28 years old, female, self employed)

These results indicate that community-level networks may both reinforce lack of trust and provide opportunities for enhancing acceptance through trusted messengers and community engagement (Burnett et al. 2019; WHO 2021).

## Discussion

This study offers qualitative findings that are context-specific and pertain to the factors that influence immunisation adherence in the Western Cape. The study has been able to identify the interrelated factors that are structural, systemic, interpersonal and cultural in nature, and therefore, the findings of the study are able to go beyond the single factor and instead focus on the strategies that can be used to improve immunisation coverage.

This research shows that childhood immunisation compliance in the Western Cape is not the result of isolated factors but is instead the result of a combination of structural, relational and contextual factors that occur at the same time. Rather than seeing non-compliance or delayed immunisation as the result of individual non-compliance, this research places the issue of compliance in a larger socio-ecological context.

Barriers to accessibility served as structural gatekeepers for care. Transport difficulties, distance, dangerous neighbourhoods and fixed clinic times cumulatively contributed to the indirect costs of accessing ‘free’ immunisation care. These factors were more than just practical nuisances and represented underlying spatial and socioeconomic disparities, as evidenced in South Africa, where infrastructure gaps were associated with disparities in immunisation rates (Ndwandwe et al. 2021; Wiysonge et al. 2021).

Notably, conflict at work highlights the struggle between health-seeking and economic survival, indicating that immunisation programs need to accommodate the realities of caregivers rather than vice versa. Flexible timing, decentralised delivery and context-specific delivery strategies thus represent equity-enhancing strategies rather than desirable service upgrades (WHO 2021).

Communication and information were revealed as relational factors that mediated trust and engagement. Poor explanation, hurried consultations and feelings of disrespect undermined caregiver confidence, while good communication supported and sustained adherence. These results confirm that immunisation practice is, in part, a product of the quality of the relationship in the consultation.

Trust in healthcare providers has been shown to be a key predictor of vaccine acceptance (Larson et al. 2020), and these results demonstrate how trust can be consolidated or undermined by everyday communication.

The preference for digital reminders also indicates a desire for reliable, supportive systems; yet innovation must remain grounded in person-centred communication to avoid the risk of technology masking relational shortcomings (Martines et al. 2023; WHO 2021).

The efficiency of the healthcare system was a real and symbolic determinant of vaccine compliance. Long waiting times, irregular appointment schedules, and vaccine shortages were perceived by caregivers as a sign of the unreliability of the healthcare system. These have implications for reputation and may serve as a deterrent for caregivers to return to the healthcare system, even if they are supportive of immunisation. The results, therefore, indicate that efficiency and reputation are interrelated. Improving the management of workflow, appointments and vaccine stability may, therefore, improve the efficiency of the healthcare system and its reputation (Burnett et al. 2019; Rodrigues, Hassan & Kumar 2024).

Parental attitudes and knowledge were characterised by a complex interplay between personal belief and social persuasion. Although many parents had incorporated immunisation as a parental duty, the influence of misinformation and negative community narratives created ambiguity. It is important to note that some participants showed resilience in fighting misinformation, which indicates the coexistence of individual agency and vulnerability. This ambivalence corresponds to the current perspective of vaccine hesitancy as a context-dependent phenomenon rather than a fixed opposition (WHO 2019). The role of provider trust as a moderator has emerged.

Socioeconomic factors did not operate as independent factors but rather as factors that increased the impact of other barriers. Financial barriers, transport challenges, insecurity of employment and safety of neighbourhoods interacted with access and system barriers to create a compound effect of disadvantage. The results are consistent with evidence that inequities in immunisation are structurally embedded and require multisectoral approaches (Martines et al. 2023; WHO 2021). It may be as important to address indirect costs as it is to address knowledge gaps.

Cultural and belief systems also impacted immunisation practices in more complex ways, through intergenerational storytelling, social influences and levels of medical distrust. Rather than being indicative of a rejection of biomedical healthcare, these factors are indicative of a form of decision-making that occurs through negotiation in a community setting. Culturally safe engagement, through partnership with respected key community leaders and recognition of community concerns, is therefore critical to long-term immunisation uptake (Burnett et al. 2019; Ndwandwe et al. 2021). Notably, the results indicate that a lack of cultural competence in healthcare can be balanced with strong public health messages.

Taken collectively, the research confirms that immunisation adherence is a multi-determined and contextually embedded process. To be effective, strategies need to go beyond the intervention and incorporate improvements in structural accessibility, trust-building, strengthening and culturally responsive communication. In terms of policy and practice in the Western Cape, the results of the research highlight the need for equity-oriented and person-centred models of immunisation that position caregivers as active decision-makers in complex social realities.

The conclusions drawn from the research indicate that adherence to vaccinations is a multi-faceted phenomenon that involves the consideration of various social and structural factors, which calls for measures to tackle not only individual aspects of immunisation, but also more general, systemic conditions. When examined within the framework of the BeSD model developed by WHO, the findings demonstrate how the complex interplay between thinking (caregivers’ knowledge and views on vaccinations), feeling (their fears associated with immunisations and health problems), social processes (such relationships as trust towards health workers and influence of the community), motivation and access (issues related to the safety, availability of transportation, and other work-related challenges) affects the immunisation process. It means that the development of intervention programmes aimed at increasing the immunisation rate requires addressing all these different aspects simultaneously. In addition, using the PARIHS framework (Promoting Action on Research Implementation in Health Services) allows determining the key factors to consider in implementing immunisation programmes in the Western Cape area. Specifically, these include the presence of relevant local evidence based on people’s experiences, supportive environment (health service responsiveness, safety and accessibility) and facilitation (health workers’ communication skills, building trust and engaging in cultural competence).

### Strengths and limitations

The qualitative aspect of this research has several strengths. Semi-structured interviews enabled the exploration of the caregivers’ perceptions in depth, and the use of thematic analysis facilitated the identification of patterns. The interviews took place in healthcare settings, ensuring relevance and grounding the findings in practice. However, there are some limitations that need to be considered. The sample, although adequate for the qualitative study, may not be representative of all caregiver groups, especially those who are totally disengaged from healthcare. Since the data were self-reported, it may have been subject to recall or social desirability bias. Despite these limitations, the qualitative results are credible and grounded in context, offering insights into immunisation adherence in the Western Cape.

### Recommendations

For better compliance with childhood immunisation in the Western Cape, what is needed is an integrated approach. Services need to improve physical accessibility by offering outreach clinics, flexible timing and possible transport assistance. Improving respectful communication and structured education of caregivers is a must for better health literacy. Electronic reminder programs and better appointment scheduling can help reduce no-shows, while better supply chain management can improve service efficiency. Finally, a multisectoral approach is required to address socioeconomic issues at the community level to ensure person-centred immunisation services.

## Conclusion

This research proves that childhood immunisation compliance in the Western Cape is influenced by interrelated structural, socioeconomic, cultural and healthcare system factors. Obstacles of access, service efficiency, communication quality, health literacy and indirect costs are responsible for the delay or missed immunisation. To overcome these issues, it is necessary to have multi-level strategies that improve service accessibility, communication quality, system performance and reduce socioeconomic limitations. By building trust and developing context-specific interventions, healthcare systems can promote more equal immunisation rates and child health outcomes.
